# Older adults with disability in extreme poverty in Peru: How is their access to health care?

**DOI:** 10.1371/journal.pone.0208441

**Published:** 2018-12-26

**Authors:** Oscar Flores-Flores, Ruth Bell, Rodney Reynolds, Antonio Bernabé-Ortiz

**Affiliations:** 1 Institute for Global Health, University College London, London, United Kingdom; 2 Universidad de San Martin de Porres, Facultad de Medicina Humana, Centro de Investigación del Envejecimiento (CIEN), Lima, Peru; 3 Universidad Cientifica del Sur, Facultad de Ciencias de la Salud, Lima, Peru; 4 UCL Institute of Health Equity, Faculty of Population Health Sciences, University College London, London, United Kingdom; 5 CRONICAS, Centre of Excellence in Chronic Diseases, Universidad Peruana Cayetano Heredia, Lima, Peru; 6 School of Medicine, Universidad Peruana de Ciencias Aplicadas–UPC, Lima, Perú; 7 Faculty of Epidemiology and Population Health, London School of Hygiene and Tropical Medicine, London, United Kingdom; Universita degli Studi di Perugia, ITALY

## Abstract

**Background:**

Disability rates increase with age. In 2012, Peruvian older adults (≥ 65 years) represented 9% of the population. Additionally, older population reported disabilities at about 5 times the rate of Peruvians between 36 and 64 years old, and 30% of older population lived in poverty. Peruvian seniors living in extreme poverty experience disabilities and the extent of their access to healthcare is unknown.

**Objective:**

This study assesses associations between disability and access to healthcare among Peruvians older individuals living in extreme poverty.

**Methods:**

Secondary analysis of a national representative population based survey that utilizes information from Peru’s 2012 survey Health and Wellbeing in Older Adults (ESBAM), which includes older adults living in extreme poverty. We define disability in terms of the Activities of Daily Living (ADL disability) framework. Healthcare access was assessed as having any of Peru’s available health insurance schemes combined with preventive health services (vision assessment, influenza vaccination, blood pressure assessment, diabetes screening, and cholesterol assessment). Poisson robust regression models were used to evaluate the associations among relevant variables. Prevalence Ratios and 95% confidence intervals (95%CI) were reported.

**Results:**

Data from 3869 individuals (65 to 80 years old), of whom 1760 (45.5%) were females, were analyzed. The prevalence of ADL disability was 17.3% (95%CI: 16.0%-18.4%). In addition, more than 60% had never received any of the preventive measures evaluated, except for the blood pressure assessment. In the adjusted model, people with ADL disability had 63% less probability of having extensive insurance, compared to those without disability (p<0.05).

**Conclusions:**

This study shows that this Peruvian older population living in extreme poverty has limited access to healthcare services. Although there was no consistent association between ADL disability and the healthcare access, there is an urgent need to reduce the inequitable access to healthcare of this poor Peruvian older population.

## Introduction

Estimates suggests that 70% of adults over age 65 live in low- and middle-income countries (LMIC), and that their number will increase in the future[1]. The accelerated ageing in LMIC goes along with the epidemiological transition, where the burden of disease shifts from communicable to non-communicable diseases (NCDs)[2]. Furthermore, in LMIC, health systems are usually precarious, and the formal social protection systems only cover a small proportion of their older population[2, 3]. In this context, with advancing age, limitations of functional capacity in performing basic activities of daily living (ADL) become more common[4]. The development of ADL limitations has deleterious impact on older adults’ live and their caregivers and significantly increase the risk of mortality[5]. For this study, we used the framework of ADL disability based on Katz index[6].

Among people with disabilities, studies have shown that they spend more money to live[7], face more challenges to find a job and suffer discrimination compared to those without disabilities[3]. Therefore, it has been proposed that the access to healthcare of persons with disabilities *“constitutes an indicator of overall equity of the healthcare system”[8].* Barriers to standard medical care and rehabilitation affect people with and without disabilities, though older adults with disabilities arguably experience greater vulnerabilities and barriers[3]. In India and South Africa, people with disabilities described barriers to access to healthcare such as cost of transportation and poor staff attitudes[9, 10]. In Brazil, older adults with disabilities encountered architectonic barriers in health services such as absence of sidewalks or ramps [11].

A middle-income country, Peru has reduced poverty and improved health outcomes as one of the Americas’ fastest-growing economies[12, 13]. Though Peru’s government recently set the goal of achieving Universal Health Coverage by 2021, fragmentation currently characterizes Peru’s health system. Five different entities administer various medical insurance schemes across the country. The Ministry of Health (MINSA), provides health services for 60% of the population; EsSalud, serves 30% of the population; the Armed Forces (FFAA), National Police (PNP), and the private sector together cover the remaining 10%[14]. MINSA offers “Comprehensive Health Insurance (SIS)”, which subsidizes medical services for people living in poverty and extreme poverty. The other schemes (FFAA, PNP and EsSalud) provide full coverage for all health necessities, including rehabilitation. SIS, by contrast, only covers a list of prioritized conditions and interventions [15]. Though the MINSA health facilities enjoy wide national distribution via Primary Health Centers (PHC), they also have few resources and limited equipment, especially in hard-to-reach rural areas. On the other hand, FFAA, PNP, EsSalud and private health facilities have greater resources and better equipment spread across fewer locations. There are national recommendations for the care of older adults, which includes the comprehensive social, mental, functional and clinical evaluation, as well as the stratification according to the frailty status and the presence of chronic diseases[16].

According to the National Institute of Statistics and Informatics (INEI, in Spanish), in 2012 the Peruvian population of adults ≥ 65 years was about 1 845 000. Of them, 75% lived in urban areas[17]. Among Peruvian seniors, 67% were affiliated to any kind of health insurance[18]. Additionally, it has been reported that more women (80%) spoke of having a chronic disease compared to men (70%)[18]. Regarding poverty, the Economic Commission for Latin America and the Caribbean (CEPAL) stated that between 2010 and 2012, 30% of older adults lived in poverty[19]. In rural area, 49% of seniors lived in poverty while in urban areas the 14% of them were in poverty[19]. Recognizing that Peruvians over the age of 65 living in poverty constitute one of the country’s most vulnerable populations, the government created a non-contributory pension to extend social protection provisions to this population. Via the “Encuesta de Salud y Bienestar del Adulto Mayor” (ESBAM) (Health and Wellbeing Survey of Older Adults), the new pension program’s initial needs evaluation collected health and well-being information about the poorest older adults. This research analysis draws on that publicly available 2012 ESBAM data.

In Peru, the results of the only nationally representative survey of disability, ENEDIS [20], show that people with disabilities experience greater (self-reported) access barriers to rehabilitation therapies than people without disabilities. Since ENEDIS reports on rehabilitation therapy and disability prevalence, and explicitly excludes those who do not need rehabilitation, it has limited utility for analysis of the broader issues of healthcare access. Nevertheless, it does confirm that a third of the population over age 65 report some disability and this group experiences multiple disabilities at about 5 times the rate of Peruvians between ages 36 and 64[9]. Our research assumes that poverty create barriers for healthcare access among Peru’s most vulnerable seniors. Accordingly, we analyzed associations between ADL disability based on Katz index and healthcare access for Peruvians age 65 and older. To describe differences in healthcare access for men and women, and between rural and urban in settings, as those were suggested as important factors [9, 21], we compared whether these variables modify health outcomes.

## Materials and methods

### Study design

This secondary analysis of a cross-sectional population based survey utilizes information from Peru’s ESBAM study. Conducted between October and November 2012, ESBAM derives its data from the Ministry of Development and Social Inclusion (MIDIS) and the Ministry of Economy and Finance’s (MEF) National Assistance Program, “Pension 65”.

### Study setting and participants

The ESBAM database collected information from individuals 65 to 80 years old who live in households under or around the extreme poverty threshold. Peru has a household focalization system (SISFOH), which is the mean for families to be eligible for social programs. SISFOH uses an algorithm that includes level of income, quality of life of the household, and expenditure of public services (if available) to determine household classification (non-poor, poor but non-extreme poor, and extreme poor)[22]. The ESBAM excluded individuals over 80 years old, who were not targets initially of the “Pension 65” program[23]. ESBAM excluded one individual with visual/auditory impairment living alone who had no caregiver to assist interviewers with data collection.

### Procedures

#### Sampling methods

12 Peruvian regions in which MIDIS had completed the census of socio-economic variables required for calculating the poverty threshold contributed data to ESBAM. It employed probabilistic sampling, independent in each region, stratified by location of residence (urban and rural) and carried out in two steps. The Primary Sampling Units (PSU) were census units in urban areas and villages in rural areas with at least 4 households living in poverty and with older individuals (≥ 65 years). The selection of PSU was made by Probability Proportional to Size (PPS) corresponding to the total number of households. Four households were randomly drawn from each PSU for interview and 2 for replacements[24]. Details regarding selection are published elsewhere[25].

#### ESBAM Questionnaire

ESBAM is Peru’s only representative survey that provides information about the wellbeing of Peruvian seniors living in extreme poverty. It includes demographic, physical and subjective health, as well as economic and social support information. Trained fieldworkers collected data face-to-face via questionnaire. None of the authors of this study collected EMBAM data.

### Definition of variables

#### Outcome

This study examines access to healthcare using the following 2 ESBAM indicators (defined below): 1) having health insurance and 2) having received specific preventive health services.

**Health Insurance:** self-reported affiliation with a health insurance scheme. Coded as: No insurance; with Comprehensive Health Insurance (SIS); and with “extensive insurance”, which includes Essalud, Private, FFAA, and PNP.

**Preventive services:** Influenza vaccination, vision examination, blood pressure and serum cholesterol assessments and diabetes screening. All Peru’s insurance types cover these.

ESBAM asked respondents for the last time they received preventative health interventions. Possible responses were: <6 months; between 6 months and <2 years; between 2 and <5 years; never. Answers were code as: “never received”, “received in a period <2 years”, and “received in a period ≥2 years”. Our study selected the ‘less than two years’ threshold for analysis, since according to medical guidelines [26–28], individuals should receive preventive interventions within this period.

#### Exposure

For this study, we used disability based on modified Katz Index for Activities of Daily Living (ADL Disability)[29]. The Katz index probes individuals’ ability to perform six activities: dressing, feeding, transferring (getting in/out of bed), walking (walking around inside), bathing, and using the toilet. In the ESBAM, interviewers asked if respondents have permanent difficulty (≥3 months) performing any of those activities and offered three possible responses: “much difficulty”; “little difficulty”; “no difficulty”. Following similar studies [30, 31], answers were collapsed into a binary variable: Having “much difficulty” and no difficulty (combined “little difficulty” and “no difficulty”). ADL disability was considered present when a person expressed “much difficulty” in any of the six daily living activities.

#### Other variables

The analysis also included other variables, potential confounders or effect modifiers on the association of interest. These included: *sex* (male vs. female); *age* (65–70, 71–75 and 76–80 years old); *area of living (*rural and urban area). Urban are those areas with at least 100 households grouped continuously, while rural are those areas with less than 100 households grouped or more than 100 households but scattered[32, 33]. *Mother tongue* as a proxy for ethnicity (Spanish, other native languages) since discrimination against ethnic minorities may impact health services access[34]; *educational level* (none, incomplete primary school, complete primary school and secondary school or over); *socioeconomic position* (this variable was created using variables related to households’ assets and facilities combined into a deprivation index[35–37], ranged score resulted between 0 and 338. Subsequently, we divided into terciles: lowest, middle, and highest); and *currently working* (yes/no).

### Data analysis

We analyzed ESBAM data using STATA 13 for Windows (StataCorp, College Station, TX, US) and described categorical variables using proportions and compared them with the Chi-squared test. Poisson crude and adjusted models using robust variance were created to assess the association between the presence of disability and the indicators of health care access. In spite of being a binary outcome, Poisson robust models were preferred instead of logistic regression as odds ratios may overestimate the strength of association between variables in cross-sectional studies[38]. To assess collinearity, variance inflation factors (VIF) were used. The adjusted model included gender, age, mother tongue, education, currently working, socioeconomic position and area. To assess the effect modification by gender or area, a likelihood test was performed. Missing data was handled by complete case analysis.

### Ethics

The ESBAM is a publicly available anonymized dataset. Therefore, approval from an Institutional Review Board was not considered mandatory. This dataset does not provide any information that would have breached participants’ confidentiality.

## Results

### Description of study population

The ESBAM survey included 4242 individuals from 12 Peruvian regions. However, we consider only 3869 (91.2%) individuals in the final sample because 80 individuals did not have information related to the exposure and 293 individuals did not have information about outcome variables. Among this sample, 1760 (45.5%) were women and the overall mean age was 71.1 (SD: 4.4) years. Most of the older adults were between 65–70 years old (49.5%). Spanish was the mother tongue in 2715 (70.2%) individuals. Regarding the level of education, 2020 (52.3%) had incomplete primary education; whereas 2360 participants lived in rural areas (61.0%) and 2675 (69.2%) reported working at the time of the interview.

### ADL Disability and associated factors

We found 17.3% ADL disability prevalence (95%CI: 16.0%–18.4%). Table 1 shows characteristics of the sample by ADL disability status. Among individuals with ADL disability, there were more participants between 76 and 80 years, with “other native languages” as a mother tongue, and without education, compared to those without ADL disability (p<0.05). Of note, a lower proportion of individuals with ADL disability reported working at the time of the survey compared to people without ADL disabilities (p <0.001), though more than 50% were actively working in both groups. The most frequent ADL disability reported was transferring (8.7%), followed by walking (6.3%)(S1 Table).

**Table 1:** Socioeconomic characteristics of the study population according to the presence of ADL disability.

	Without ADL disability (n = 3202)	With ADL disability (n = 667)	
	N (%)	N (%)	*P value*
**Gender (n = 3 869)**			**0.009**
Female	1426 (44.5)	334 (50.1)	
Male	1776 (55.5)	333 (49.3)	
**Age (n = 3 869)**			**<0.001**
65–70 years	1626 (50.8)	287 (43.0)	
71–75 years	985 (30.8)	219 (32.8)	
76–80 years	591 (18.4)	161 (24.1)	
**Mother tongue (n = 3 868)**			**0.02**
Spanish	2273 (71.1)	442 (66.3)	
Others languages	928 (28.9)	225 (33.5)	
**Educational Level (n = 3 863)**			**0.01**
None	844 (26.4)	209 (31.4)	
Incomplete Primary	1676 (52.4)	344 (51.6)	
Complete Primary	449 (14.1)	81 (12.2)	
Secondary and above	228 (7.1)	32 (4.8)	
**Socioeconomic Position (n = 3 869)**			**<0.001**
Lowest	1388 (43.4)	243 (36.4)	
Middle	733 (22.9)	206 (30.9)	
Highest	1081 (33.8)	218 (32.7)	
**Area of Living (n = 3869)**			0.92
Rural	1952 (61.0)	408 (61.2)	
Urban	1250 (39.0)	259 (38.8)	
**Currently working (n = 3868)**			**<0.001**
No	885 (27.6)	308 (46.3)	
Yes	2317 (72.4)	358 (53.7)	

Result may not add due to missing values. Percentages were calculated in columns. Bold *p* values were significant (p<0.05).

### Access to health care: Health insurance and preventive services

In relation to health insurance, 2361 (61.0%) individuals reported having SIS, 99 (2.6%) having extensive insurance and 1409 (36.4%) reported no insurance. Four individuals had ≥2 kinds of health insurance and so were excluded for further analysis. Overall, the proportion of women with any kind of insurance was 64.8%, while the proportion of men with insurance was 62.7%. Table 2 shows socioeconomic characteristics according to the possession of health insurance. A larger proportion of people with languages other than Spanish did not have any insurance (p<0.05). People with secondary education or above had more access to “extensive insurance”, as did individuals living in urban areas (p<0.05).

**Table 2:** Socioeconomic characteristics of the study population by health insurance.

	Without any health insurance (n = 1 409) N (%)	With SIS insurance (n = 2 361) N (%)	With other health insurances (n = 99) N (%)	*P* *value*
**Gender (n = 3 869)**				0.40
Female	621 (44.1)	1 092 (46.3)	47 (47.5)	
Male	787 (55.9)	1 269 (53.7)	52 (52.5)	
**Age (n = 3 869)**				0.48
65–70 years	724 (51.4)	1 143 (48.4)	46 (46.5)	
71–75 years	421 (29.9)	750 (31.8)	33 (33.3)	
76–80 years	263 (18.7)	468 (19.8)	20 (20.2)	
**Mother tongue (n = 3 868)**				0.856
Spanish	990 (70.3)	1 658 (70.2)	67 (67.7)	
Others languages	418 (29.7)	703 (29.8)	32 (32.3)	
**Educational Level (n = 3 863)**				**<0.001**
None	357 (25.4)	679 (28.8)	17 (17.4)	
Incomplete Primary	706 (50.2)	1275 (54.1)	39 (39.8)	
Complete Primary	233 (16.6)	284 (12.0)	13 (13.2)	
Secondary and above	110 (7.8)	121 (5.1)	29 (29.6)	
**Socioeconomic Position (n = 3 869)**				**<0.001**
Lowest	559 (39.7)	1069 (45.2)	3 (3.1)	
Middle	292 (20.8)	632 (26.8)	14 (14.1)	
Highest	557 (39.5)	660 (28.0)	82 (82.8)	
**Area of living (n = 3 869)**				**<0.001**
Rural	764 (54.3)	1585 (67.1)	10 (10.1)	
Urban	644 (45.7)	776 (32.9)	89 (89.9)	
**Currently working (n = 3 868)**				0.33
No	426 (30.2)	730 (30.9)	37 (37.4)	
Yes	982 (69.8)	1630 (69.1)	62 (62.6)	
**ADL Disability (n = 3 869)**				**0.005**
No	1176 (83.5)	1933 (81.9)	93 (93.9)	
Yes	233 (16.5)	428 (18.1)	6 (6.1)	

Result may not add due to missing values. Percentages were calculated in columns. Four people that had both categories of health insurance were excluded from the analysis. Bold p values were significant (p<0.05)

Regarding the preventive service of flu vaccination, 3050 (78.8%) reported never having received it. In relation to screening activities: 2531 (65.4%) had never had a vision evaluation, 1320 (34.1%) never had had a blood pressure assessment; 2522 (65.2%) never had had a serum cholesterol assessment; and finally, 2924 (75.6%) never had had a diabetes screening. S2 Table shows the socioeconomic characteristics of the study population by preventive assessments.

### Association between ADL disability and access to healthcare

The multivariable model shows evidence of an association between ADL disability and access to “extensive insurance”: individuals with ADL disability had a 63% lower probability of having this kind of insurance when compared to individuals without ADL disability (Table 3).

**Table 3:** Association between disability and having health insurance.

Disability	SIS (vs. no insurance)		Other insurances (vs. no insurance)	
	Crude model	Adjusted model*	Crude model	Adjusted model*
	PR (95%CI)	PR (95%CI)	PR (95%CI)	PR (95%CI)
No	1 (Reference)	1 (Reference)	1 (Reference)	1 (Reference)
Yes	1.04 (0.98–1.11)	1.03 (0.97–1.09)	**0.34 (0.15–0.77)**	**0.37 (0.16–0.85)**
***Among men***				
No	1 (Reference)	1 (Reference)	1 (Reference)	1 (Reference)
Yes	**1.12 (1.03–1.22)**	**1.11 (1.02–1.20)**	0.71 (0.28–1.73)	0.86 (0.35–2.15)
***Among women***				
No	1 (Reference)	1 (Reference)	1 (Reference)	1 (Reference)
Yes	0.95 (0.87–1.05)	0.95 (0.86–1.04)	**0.09 (0.13–0.65)**	**0.09 (0.12–0.68)**
***In rural areas***				
No	1 (Reference)	1 (Reference)	1 (Reference)	1 (Reference)
Yes	1.04 (0.97–1.12)	1.03 (0.96–1.11)	2.22 (0.58–8.50)	1.57 (0.34–7.13)
***In urban areas***				
No	1 (Reference)	1 (Reference)	1 (Reference)	1 (Reference)
Yes	1.05 (0.93–1.17)	1.01 (0.90–1.14)	**0.19 (0.06–0.59)**	**0.20 (0.06–0.65)**

PR: Prevalence Ratio.

*Adjusted by gender, age, education, area of living, and socioeconomic position. Bold values are significant (p<0.05)

Sex was an effect modifier of the association between ADL disability and health insurance (both, SIS and “extensive insurance”) (p<0.05): among men, those individuals with ADL disability had an 11% more probability of having SIS insurance compared to those without ADL disability. On the other hand, among women, individuals with ADL disability had a 91% lower probability of having “extensive insurance”. In addition, area of living was an effect modifier of the association between disability and having “extensive insurance” (p<0.05): among urban residents, those individuals with ADL disability had an 80% lower probability of having “extensive insurance” compared to those without disabilities. The association was not present in the people who live in rural area.

When the association between preventive activities and ADL disability was assessed (Table 4), only having had a blood pressure assessment was significant: compared to individuals without ADL disability, those with ADL disability had a 10% more probability of having had blood pressure assessment within the previous 2 years of the survey, after controlling for potential confounders. When interaction was explored, area of living was an effect modifier of the association between ADL disability and vision and blood pressure assessment (p<0.05): among rural dwellers, those individuals with ADL disability had a 32% (95%CI: 3%- 69%) and 22% (95%CI: 11%-34%) greater probability of having vision assessment and blood pressure measurement within the previous 2 years, respectively, compared to those without disabilities. The association was not present in the urban group in the association between disability and vision assessment (p = 0.30).

**Table 4:** Association between disability and preventive activities.

Disability	<2 years (vs. Never)		≥2 years (vs. Never)	
	Crude model	Adjusted model*	Crude model	Adjusted model*
	PR (95%CI)	PR (95%CI)	PR (95%CI)	PR (95%CI)
	***Influenza vaccination***			
No	1 (Reference)	1 (Reference)	1 (Reference)	1 (Reference)
Yes	1.14 (0.94–1.38)	1.13 (0.93–1.37)	0.86 (0.62–1.19)	0.86 (0.62–1.20)
	***Vision assessment***			
No	1 (Reference)	1 (Reference)	1 (Reference)	1 (Reference)
Yes	1.09 (0.92–1.29)	1.07 (0.90–1.27)	1.14 (0.96–1.35)	1.10 (0.93–1.31)
	***Blood pressure measurement***			
No	1 (Reference)	1 (Reference)	1 (Reference)	1 (Reference)
Yes	**1.10 (1.03–1.18)**	**1.10 (1.02–1.17)**	1.14 (0.96–1.37)	1.09 (0.92–1.31)
	***Serum cholesterol measurement***			
No	1 (Reference)	1 (Reference)	1 (Reference)	
Yes	1.06 (0.92–1.23)	1.05 (0.91–1.22)	0.96 (0.77–1.20)	0.93 (0.76–1.16)
	***Diabetes Screening***			
No	1 (Reference)	1 (Reference)	1 (Reference)	1 (Reference)
Yes	1.14 (0.95–1.36)	1.13 (0.94–1.34)	0.84 (0.62–1.14)	0.80 (0.60–1.08)

PR: Prevalence Ratio.

*Adjusted by gender, age, education, area of living, and socioeconomic position. Bold values are significant (p<0.05)

## Discussion

### Main findings

Almost forty percent of Peru’s older adults living in extreme poverty lacked health insurance. Moreover, ADL disability based on Katz index was associated with lower probability of having extensive health insurance (i.e. FFAA, PNP, EsSalud & private insurance), which provides services with greater resources and technology than the SIS insurance scheme, but requires contributory payments[15]. Among men, having ADL disability increased the probability of having SIS insurance.

At the same time, more than 60% of the population had never accessed preventive health services, with the exception of blood pressure assessments. Additionally, in rural areas, having ADL disability increased the probability of having had blood pressure measurement and vision assessment in the previous 2 years.

### Prevalence of ADL disability

Our analysis found lower prevalence of ADL disability among Peru’s poorest and oldest people (17%) when compared with Santiago de Chile’s (34%) and Buenos Aires’ (32%) populations over age 75[39]. In Chile and Argentina, broader definitions of ADL disability than those employed in our study help explain this variance. In our analysis, in order to select the population most affected by disability, we determined ADL disability as congruent with responses that signaled “much difficulty”. If we were to define ADL differently or combine “much difficulty” and “some difficulty”, the sample prevalence of ADL would increase to 56%.

Consistent with several studies [29, 40, 41], falling into the oldest age grouping, not working and lacking education were factors associated with having greater ADL disability. Nevertheless, it is important to note that in spite of being an older population (mean age = 71.1 years), most of them continue working (>50%), probable because they, as being a poor population, need to get some income to subsist day by dedicated to agriculture or small trade business[42]. We also identified an association between having other languages as a mother tongue and positive ADL disability. Peru’s history of social and spatial discrimination practices lead us to interpret this finding to suggest that since childhood, indigenous speakers likely experienced greater isolation and poorer health services utilization[43].

### Access to health care and ADL disability

#### Health insurance

The World Report of Disability[3] emphasizes that having insurance can increase access to, and use of health care services among people living with disability. Insurance may also improve a variety of outcomes including an increase in the probability of receiving primary care, a decrease in unmet needs, and a reduction in delays. In our analysis, for individuals with SIS insurance, we identify no healthcare access difference regardless of ADL disability status. However, 36% of those surveyed reported no insurance, though they qualified for it. This result reflects poor distribution of health system resources. Medical services never reached this group removing their opportunity to utilize health insurance for their care needs.

Individuals with ADL disability had a lower probability of having “extensive insurance” than those without ADL disability. Having access to “extensive insurance” depends on factors such as former or current formal employment, having children who can contribute economically or who were members of the Police or Military Forces. The most probable explanation for the finding is that people with ADL disability are poorer in comparison to those who do not have ADL disability. Individuals with ADL disability make up a smaller proportion of people who are currently working and obtaining an income. Furthermore, since ESBAM analyzes cross-sectional data, the impact of duration of living in poverty on disability and vice versa is unknown. People living in “chronic” poverty are more likely to become ill and not obtain access to proper care, and over time experience complications and disability[44]. In our analysis, we adjusted the association between ADL disabilities and “extensive insurance” by a socioeconomic position (SES) variable; however, this variable was based on assets and basic household fixtures and we were unable to include the financial contribution that children may provide, resulting in the possibility of residual confounding.

Among men, having ADL disability was associated with a higher probability of obtaining SIS insurance. A possible explanation for this finding is that Peruvian males do not find affiliating to SIS insurance relevant nor necessary when they are fit enough to work[45]. Nevertheless, when they present a disability, they or their families look for health services in order to recover their independence.

Urban residents with ADL disabilities obtain “extensive insurance” less frequently compared to urban residents without disabilities. One explanation related to poverty could be the environmental factors that cites have (services, attitudes, accessibility of physical environment) that limits considerable the possibility to work, and consequently, obtaining some income to access this type of insurance.

#### Preventive services

All preventive services (vaccination and the four assessments) had very low levels of coverage. Interestingly, most respondents (60%) never received preventive health interventions (except for blood pressure assessment), although 60% of this population claimed health insurance. Such findings raise concerns about how possessing health insurance increases (preventative) healthcare access. It is possible that health insurance increases curative and rehabilitation services use [20], but has limited preventive care utilization.

Health systems in Latin America were developed and organized in a context of economic crisis, leading to a fragmentation of the health system which complicates the delivery of services[46]. Primary care facilities find wide distribution across Peru, especially through MINSA, and should emphasize preventive care. However, these facilities experience economic, technological and human resources shortages, reflecting the precarious primary health care in Peruvian health system[46]. Additionally, Peru is one of the countries with the lowest investment in health sector, (4.8% GDP) which prevents to the delivery, management and informatics systems from being properly upgrade[47]. Furthermore, health professionals are trained mainly in schools that focus on recuperative services, perpetuating the willingness to curative approach.

In the case of vision assessment, although several causes of blindness can be prevented (cataracts, refraction problems), access to screening services was less than 40%. Surprisingly, living with ADL in rural areas increased the probability of obtaining this assessment. The reason for this may be related to the launch of the national plan to fight cataracts (2007–2010) by the Minister of Health[48] and presumably due to the awareness this program generated about visual health. As a result, campaigns for the prevention and detection of cataracts have increased across the country. Additionally, in rural areas, these campaigns are the only opportunity rural residents have for obtaining a cataract operation or for acquiring adequate eyeglasses. As a result, individuals actively seek these campaigns.

Regarding Influenza vaccinations, in the ESBAM sample, the coverage of influenza vaccination in the previous 2 years was markedly low (<20%). Comparatively, national samples from other regions showed notably higher vaccination coverage: 73% of older adults were reached in Colombia [49] whereas in Mexico [50], coverage was 40% higher than the reported ESBAM sample. From the perspective of service provision, vaccination programs require greater logistical organization than other kinds of interventions and so face challenges such as cold storage of vaccines, secure transportation, etc. More importantly, the target population for influenza vaccination includes not only the seniors but also children and pregnant women. Therefore, in some areas, there may be a competition for supplies when there are limited quantities of vaccines available. On the other hand, older adults may not actively seek vaccination, because the need for it is less obvious to them, in comparison to vision assessment.

Blood pressure measurement was the assessment with the best coverage among this population with respect to preventative health interventions. Moreover, having ADL disability in rural areas increased the probability of access to this assessment. The higher rate of blood pressure monitoring may be explained by the lack of logistical barriers that could interfere with taking blood pressure measurements. The sphygmomanometer is a widely distributed instrument in every health facility, even in remote rural areas. Thus, people with ADL disability, which may be secondary to a subjacent disease, might be looked periodically for health care and what they obtained was measurement of blood pressure as a part of the usual triage (blood pressure measurement, weight, and temperature). Although the question about blood pressure measurements refers to preventive services, interviewers may have misunderstood that and answered according to whether they received blood pressure assessment or not during a doctor’s triage appointment.

### Study Implications

Though it was expected that people with ADL would have a consistently lower level of access to the selected indicators of health care, this study shows that irrespective of the ADL disability status, a great majority of the study population lacks basic healthcare. That does not discourage the importance of including disability in a model of healthcare in order to promote equity and social justice. Furthermore, the Peruvian government should increase its efforts to improve the conditions that will allow utilization of health insurance. However, increasing SIS coverage must accompany improved services distribution and quality. The fact that only the preventive services that do not require extensive logistics are able to reach this population, highlights the necessity for upgrading the services delivery capability of the health system.

### Strengths and Limitations

Despite the pressing need for data and analysis about social exclusion and access to health care, evidence relating to these issues is still lacking in many LMICs. Therefore, this study is pertinent as it employs an equity approach, focusing on one of the most vulnerable and excluded populations in Peru. However, this study also has some limitations. First, possibly people with most severe disabilities were not included, which could skew associations between ADL disability and healthcare access. Additionally, it is important to note that the tool developed by the Washington Group to collect information about disability is the preferred method worldwide[51]. However, the limitations of the dataset prevents us from using this and we focused our study on functional disability measured by the modified Katz scale. Second, since ESBAM uses self-reported information, recall bias may affect reporting of outcomes (i.e. report of preventative health interventions during the previous year), especially given the age of the study population. Nevertheless, they did not include people over 80 years to reduce recall bias. Moreover, lack of access to healthcare could potentially lead to less awareness of ADL limitations. The third is selection bias as more males were recruited in the sample. This may be due women moving to live with their children once their partner dies and therefore not being present to enroll in the study. Another reason is the exclusion of older over 80 years old, who might be the predominately female group. However, these hypotheses requires further exploration. Fourth, though we tried to adjust the models for the most important confounders, the ESBAM data does not have accurate questions related to chronic diseases, including depressive symptoms. Finally, it is also possible that the likelihood test does not have enough power to determine whether sex and area of living are effect modifiers in the associations evaluated.

## Conclusions

There were no consistent associations between ADL disability based on Katz index and the indicators of access to healthcare. Having ADL disability was associated with less access to healthcare only for “extensive insurance”. The study shows that this Peruvian population over the age of 65, irrespective of their ADL disability status, have limited access to healthcare and they do not receive many preventive health services. There is an urgent need to reduce the inequitable access to healthcare of this poor Peruvian population.

## Supporting information

S1 Table Frequency of ADL disabilities. (DOCX)

S2 Table Socioeconomic characteristics of the study population by preventive assessments. (DOCX)

## Abbreviations

ADL: Activity of Daily Living
ESBAM: Health and Wellbeing of Older Adults Survey
MIDIS: Ministry of Development and Social Inclusion
SIS: Comprehensive Health Insurance
LMIC: Low-and-middle income countries
MEF: Ministry of Economy and Finances
PSU: Primary Sampling Units
PPS: Probability Proportional to Size
STATA: Data Analysis and Statistical Software for Professionals
VIF: variance inflation factors
