# Supplementary material for: Older adults with disability in extreme poverty in Peru: How is their access to health care?
Source: PLoS One. 2018 Dec 26;13(12):e0208441. doi: 10.1371/journal.pone.0208441 (PMC6306199; doi:10.1371/journal.pone.0208441)
Supplement: S1 Table — (DOCX) [file pone.0208441.s001.docx]

**S1- Table1**

Frequency of ADL disabilities

|  | **Women** | | **Men** | |  | ***Total (%)*** |
| --- | --- | --- | --- | --- | --- | --- |
| **ADL disabilities** | Yes  N (%) | No  N (%) | Yes  N (%) | No  N (%) | ***P value*** |  |
| Bathing | 94 (5.3) | 1665 (94.7) | 95 (4.5) | 2012 (95.5) | 0.2 | 189 (4.9%) |
| Feeding | 97 (5.5) | 1659 (94.5) | 104 (4.9) | 2000 (95.1) | 0.42 | 201 (5.2%) |
| Walking | 122(6.9) | 1638 (93.1) | 123 (5.8) | 1986 (94.2) | 0.16 | 245 (6.3%) |
| Transferring | 175 (9.9) | 1583 (90.1) | 160 (7.6) | 1947 (92.4) | **0.009** | 335 (8.7%) |
| Toileting | 121 (6.9) | 1638 (93.1) | 122(5.8) | 1987 (94.2) | 0.16 | 243 (6.3%) |
| Dressing | 82 (4.7) | 1677 (95.3) | 62 (2.9) | 2042 (97.1) | **0.005** | 144 (3.7%) |
