# Supplementary material for: Older adults with disability in extreme poverty in Peru: How is their access to health care?
Source: PLoS One. 2018 Dec 26;13(12):e0208441. doi: 10.1371/journal.pone.0208441 (PMC6306199; doi:10.1371/journal.pone.0208441)
Supplement: S2 Table — (DOCX) [file pone.0208441.s002.docx]

**S2 Table**

Socioeconomic characteristics of the study population by preventive assessments

|  | | **Vision**  **Assessment** | | **Influenza**  **vaccination** | | **Blood Pressure Measurement** | | **Serum Cholesterol measurement** | | **Diabetes**  **Screening** | |
| --- | --- | --- | --- | --- | --- | --- | --- | --- | --- | --- | --- |
|  | **N** | **Never** | **<2 years** | **Never** | **<2 years** | **Never** | **<2 years** | **Never** | **<2 years** | **Never** | **<2 years** |
| **Gender** |  |  |  |  |  |  |  |  |  |  |  |
| Female | 1760 | 64.3% | 17.4% | 78.3% | 14.9% | **31.1%** | **54.9%** | **62.1%** | **25.0%** | **72.3%** | **18.3%** |
| Male | 2109 | 66.4% | 18.7% | 79.3% | 14.0% | **36.6%** | **48.4%** | **67.8%** | **19.9%** | **78.3%** | **14.5%** |
| **Age** |  |  |  |  |  |  |  |  |  |  |  |
| 65-70 years | 1913 | **68.2%** | **16.9%** | 79.3% | 13.3% | 36.2% | 49.6% | 66.8% | 21.4% | 77.5% | 15.2% |
| 71-75 years | 1204 | **65.1%** | **17.2%** | 77.7% | 16.4% | 32.0% | 53.2% | 63.3% | 22.3% | 73.8% | 17.4% |
| 76-80 years | 752 | **58.8%** | **21.3%** | 79.6% | 14.1% | 32.4% | 53.0% | 64.1% | 24.0% | 73.7% | 17.0% |
| **Mother tongue** |  |  |  |  |  |  |  |  |  |  |  |
| Spanish | 2715 | **64.4%** | **17.9%** | **78.9%** | **15.1%** | **27.5%** | **58.0%** | **60.9%** | **25.6%** | **72.0%** | **18.8%** |
| Others languages | 1153 | **67.0%** | **17.6%** | **78.8%** | **12.8%** | **49.6%** | **35.7%** | **75.2%** | **14.3%** | **84.0%** | **10.3%** |
| **Education level** |  |  |  |  |  |  |  |  |  |  |  |
| None | 1 053 | **74.3%** | **12.6%** | 78.9% | 14.5% | **36.8%** | **49.7%** | **67.5%** | **19.9%** | **78.5%** | **13.7%** |
| Incomplete Primary | 2 020 | **65.3%** | **17.7%** | 79.5% | 13.9% | **33.8%** | **52.7%** | **66.4%** | **21.7%** | **76.2%** | **16.4%** |
| Complete Primary | 530 | **57.2%** | **23.6%** | 75.7% | 16.0% | **32.5%** | **48.5%** | **62.5%** | **24.9%** | **71.9%** | **17.6%** |
| Secondary and above | 260 | **48.5%** | **28.1%** | 79.6% | 13.9% | **29.6%** | **53.9%** | **52.7%** | **29.2%** | **67.3%** | **21.5%** |
| **Socioeconomic Position** | |  |  |  |  |  |  |  |  |  |  |
| Lowest | 1 631 | **75.8%** | **12.4%** | 79.0% | 14.6% | **41.3%** | **45.2%** | **75.8%** | **14.2%** | **83.8%** | **10.4%** |
| Middle | 939 | **67.4%** | **16.0%** | 79.6% | 13.2% | **36.0%** | **48.3%** | **69.6%** | **18.9%** | **79.7%** | **12.5%** |
| Highest | 1 299 | **51.0%** | **26.0%** | 77.8% | 14.9% | **23.8%** | **61.4%** | **48.7%** | **34.7%** | **62.2%** | **26.4%** |
| **Area of living** |  |  |  |  |  |  |  |  |  |  |  |
| Rural | 2 360 | **73.4%** | **13.3%** | 78.9% | 14.5% | **40.4%** | **45.4%** | **73.8%** | **15.1%** | **83.1%** | **10.1%** |
| Urban | 1 509 | **52.9%** | **25.0%** | 78.8% | 14.3% | **24.3%** | **60.7%** | **51.6%** | **33.3%** | **63.8%** | **26.0%** |
| **Currently working** |  |  |  |  |  |  |  |  |  |  |  |
| No | 1 193 | **61.8%** | **19.2%** | 78.5% | 15.8% | **28.3%** | **59.1%** | **56.7%** | **29.3%** | **68.2%** | **21.5%** |
| Yes | 2 675 | **67.0%** | **17.3%** | 79.0% | 13.8% | **36.8%** | **47.9%** | **69.0%** | **19.0%** | **78.9%** | **14.0%** |
| **Disability** |  |  |  |  |  |  |  |  |  |  |  |
| No | 3 202 | 66.0% | 17.7% | 79.0% | 14.0% | **35.1%** | **50.5%** | 65.3% | 21.9% | 75.7% | 15.8% |
| Yes | 667 | 62.8% | 18.7% | 78.0% | 16.2% | **29.7%** | **55.5%** | 64.5% | 23.5% | 74.8% | 18.3% |
| Proportion of individuals who never received the assessment or received it within a period less than 2 years is shown. Percentages were calculated in rows. Bold number are significant at the p<0.05 level. | | | | | | | | | | | |
